# Handgrip strength is associated with risks of new-onset stroke and heart disease: results from 3 prospective cohorts

**DOI:** 10.1186/s12877-023-03953-8

**Published:** 2023-05-04

**Authors:** Guochen Li, Yanqiang Lu, Liping Shao, Luying Wu, Yanan Qiao, Yi Ding, Chaofu Ke

**Affiliations:** 1 Department of Epidemiology and Biostatistics, School of Public Health, Medical College of Soochow University, 215123 Suzhou, P. R. China; 2 Department of Preventive Medicine, College of Clinical Medicine, Suzhou Vocational Health College, 215009 Suzhou, P. R. China

**Keywords:** Handgrip strength, Cohort study, Stroke, Heart disease

## Abstract

**Background:**

Stroke and heart disease are two major contributors to the global disease burden. We aimed to evaluate and compare the roles of different handgrip strength (HGS) expressions in predicting stroke and heart disease in three nationally representative cohorts.

**Methods:**

This longitudinal study used data from the Health and Retirement Study (HRS), the Survey of Health, Ageing, and Retirement in Europe (SHARE), and the China Health and Retirement Longitudinal Study (CHARLS). The Cox proportional hazard model was applied to analyze the relationship between HGS and stroke and heart disease, and Harrell’s C index was used to assess the predictive abilities of different HGS expressions.

**Results:**

A total of 4,407 participants suffered from stroke and 9,509 from heart disease during follow-up. Compared with the highest quartile, participants in the lowest quartile of dominant HGS, absolute HGS and relative HGS possessed a significantly higher risk of new-onset stroke in Europe, America, and China (all *P* < 0.05). After adding HGS to office-based risk factors, there were minimal or no differences in the increases of Harrell’s C indexes among three HGS expressions. In contrast, the modest association between HGS and heart disease was only seen in SHARE and HRS, but not in CHARLS.

**Conclusion:**

Our findings support that HGS can be used as an independent predictor of stroke in middle-aged and older European, American and Chinese populations, and the predictive ability of HGS may not depend on how it is expressed. The relationship between HGS and heart disease calls for further validation.

**Supplementary Information:**

The online version contains supplementary material available at 10.1186/s12877-023-03953-8.

## Background

Cardiovascular diseases (CVDs), principally stroke and heart disease, are the major contributors to the global disease burden [1]. According to the latest data from the American Heart Association, approximately 19 million deaths were attributed to CVD globally in 2020, which amounted to an increase of 18.7% from 2010 [2]. Meanwhile, in the two decades since 1996, cardiovascular disease spending in the US adult increased by >$100 billion [3]. Even in low- and middle-income countries, the average monthly treatment costs for stroke and coronary heart disease (CHD) were estimated to reach $300 and $1,000, respectively [4]. These unfavorable statistics highlight that cardiovascular diseases remain a major public health issue, which calls for effective prevention and control measures.

Handgrip strength (HGS), as a tool to reflect muscle strength, can be measured by a simple and budget-friendly method. Prior evidence indicates that the release of myokines from skeletal muscle preserves or augments vascular function [5], and low HGS or muscle strength could warn of cardiovascular risk markers such as circumference, fat mass and high-sensitivity C-reactive protein [6]. Therefore, it is reasonable to spectacle that HGS may associate with risks of stroke and heart disease. However, although HGS has been closely linked with the incidence and mortality of cardiovascular diseases in many studies [7–11], the results were not totally consistent [12–14]. For example, during a 4-year follow-up, the Prospective Urban Rural Epidemiology (PURE) study showed a significant association between HGS and the risk of stroke and myocardial infarction [11]. In contrast, a Japanese study supported that the association between HGS and cardiometabolic risk only occurred in women [12], and another study suggested that the association was only seen in men [13]. More recently, the CoLaus population-based study found that the association between low HGS and incident cardiovascular events was no longer significant after adjusting for baseline education level, job position, waist-to-hip ratio and height [14]. Additionally, different forms of HGS measurements were applied in these studies, such as dominant HGS (maximal HGS of dominant hand), absolute HGS (the sum of maximal HGS of both hands) and relative HGS (absolute HGS divided by body mass index). Several studies have suggested that relative HGS is superior to dominant HGS in capturing conceptual concomitant health and cardiovascular risk [15–17]. However, there is no general consensus in this regard. Therefore, the real associations between HGS and stroke and heart disease and whether this relation differs due to different HGS expressions remain to be clarified.

In this study, we investigated the longitudinal associations between HGS and stroke and heart disease using three prospective cohorts. In addition, we assessed and compared the predictive values of different HGS expressions on stroke and heart disease.

## Materials and methods

### Study design and population

We used data from the Survey of Health, Ageing, and Retirement in Europe (SHARE), the Health and Retirement Study (HRS), and the China Health and Retirement Longitudinal Study (CHARLS). SHARE surveyed individuals over age 50 and their partners or spouses in 29 European countries since 2004, with the aim of providing an overview of aging in Europe [18]. The HRS is the largest ongoing nationally representative longitudinal survey of all older Americans in the United States. The cohort began in 1992 and included over 22,000 adults over the age of 50 years at baseline with follow-up occurring every two years. Since 2006, HRS data collection has been expanded to include biomarkers and genetics, as well as deeper psychological and social backgrounds [19]. CHARLS surveyed Chinese people over the age of 45 and their spouses since 2011, including assessing the social, economic, and health status of community residents [20]. The three studies were approved by the corresponding ethics review committees, and all participants signed an informed consent form.

**Fig. 1:**
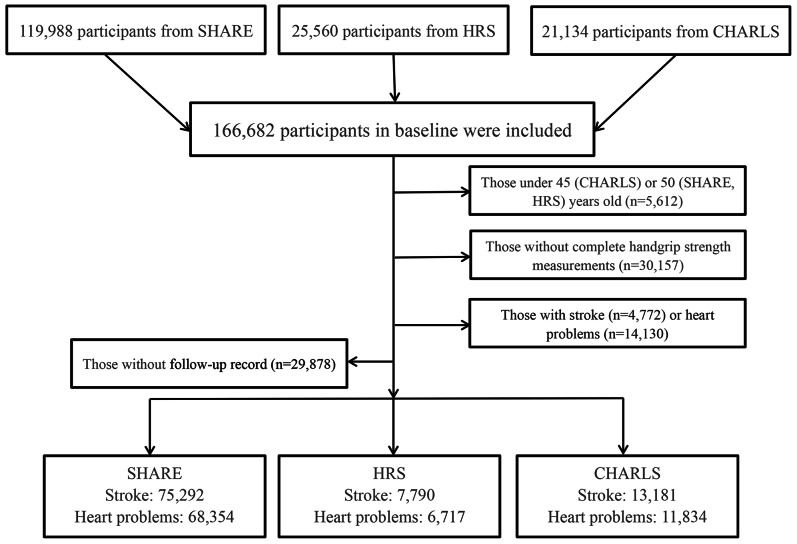
Flowchart of the selection of the study population

Participants from the three dynamic cohorts were excluded in the analysis if they met any of the following criteria: (1) age under 45 (CHARLS) or 50 (SHARE, HRS) at baseline; (2) with missing information on stroke or heart diseases at baseline; (3) not successful measurement of HGS at baseline; (4) complete loss of follow-up record and (5) suffered from stroke or heart disease at baseline. The details of the selection process can be found in Fig. 1.

## Measurement of HGS

HGS was measured by a handheld dynamometer (Smedley, S Dynamometer, TTM, Tokyo, 100 kg or WCS-100, Nantong, China) in the three cohorts at baseline [21–23]. The participants were asked whether they were in a safe state in which the measurement could be done before initiation. If the respondents refused to accept the measurement or had surgery, any swelling, inflammation, severe pain, or injury on one or both hands in the past six months, no measurement was performed. The test was performed in a standing or sitting position with elbows at 90° angles on both sides. Keep the wrist in a neutral position and adjust the internal lever of the dynamometer to fit the hand.

In this study, the body mass index (BMI, kg/m^2^) was calculated as the weight (kg) divided by the square of height (m). The dominant HGS was defined as the maximal HGS of the dominant hand, and the absolute HGS was calculated as the summation of the maximal recording from each hand. By contrast, the relative HGS was defined as the absolute HGS divided by BMI [24]. All HGS data we used were measured at baseline when participants entered the study.

## Assessment of outcomes and onset time

The diagnosis of stroke and heart disease was determined by the information self-reported by the participants in the follow-up survey. In each wave of the follow-up survey, the physician asked if he/she had had or currently had stroke and heart disease (including myocardial infarction, coronary thrombosis and any other heart problems). If an affirmative answer was given, they were regarded as new-onset disease cases. We determined the onset time as the age first diagnosed with stroke or heart disease; if this information was not available, we used the midpoint of the interval between the latest wave with available diagnostic information and the baseline wave as the onset time [25, 26].

## Covariates

The covariates in the current study were extracted from the baseline waves, including age (years), gender (man/woman), education level (less than upper secondary education, upper secondary and vocational education, tertiary education in SHARE and CHARLS, LT high-school, general educational development, high-school graduate, some college, college and above in HRS), residence (rural/urban). In addition, drinking status was defined as alcoholic drink weekly or during the last 7 days (yes or no) in SHARE, number of days of drinking per week in HRS and current drinking (yes or no) in CHARLS. Smoking status was defined as current smoking (yes or no) in SHARE, HRS and CHARLS. Moreover, the body mass index was adjusted per standard deviation (SD). In addition, the common disease conditions were also investigated at baseline by asking: “Have you been diagnosed with the following diseases by a doctor: hypertension/cancer/lung disease/ diabetes/any emotional, nervous, or psychiatric problems?”. These common diseases were adjusted for in the multifactorial model, respectively. All covariates were measured at baseline at the same time with HGS measurement.

### Statistical analysis

Categorical variables were presented by frequency (percentage). Continuous data were described as mean (standard deviation, SD). The three types of HGS indicators (dominant, absolute and relative HGS) were analyzed as both categorical variables (gender-specific quartiles, Q1-Q4) and continuous variables (per SD). The ranges for gender-specific quartiles in the 3 cohorts were showed in Supplemental Table 1.

In the main analysis, the relationship between HGS and cardiovascular disease was examined in the following two stages. First, we estimated the hazard ratios (HRs) and 95% confidence intervals (95% CIs) for the associations between HGS indicators and cardiovascular disease in each cohort study using the Cox proportional hazards regression model. The adjusted covariates included age, gender, residence, education level, BMI (not adjusted in relative HGS analysis), smoking status, drinking status, and other chronic diseases. In addition, to assess the predictive ability of HGS indicators, we calculated Harrell’s C index change upon the addition of HGS to an office-based risk score (age, gender, BMI, diabetes, hypertension, and smoking) [27, 28].

Moreover, considering death (without experiencing stroke or heart disease) as a competing risk, the Fine-Gray competitive risk model was also used to examine the associations of HGS with stroke and heart disease and compute the subdistribution hazards ratios. Finally, we also performed subgroup analyses by gender (men or women) and age (< 60 or ≥ 60 years) to assess the robustness of the associations.

Schoenfeld residuals were tested to verify the proportional risk assumption. Statistical analysis was performed using SAS 9.4 (SAS Institute Inc, Cary, NC, USA) and R 4.0.3. Two-sided *P* values less than 0.05 were deemed statistically significant.

## Results

### Results from SHARE

In total, 75,292 individuals (mean age of 63.7 years, 54.0% women) and 68,354 individuals (mean age of 63.1 years, 55.1% women) were included in the “stroke” and “heart disease” studies, respectively. With a median follow-up time of 4.67 and 4.25 years, 3,646 participants suffered from stroke and 7,552 from heart disease (Table 1).

**Table 1:** Characteristics of the study population

		SHARE			HRS			CHARLS	
		Stroke	Heart disease		Stroke	Heart disease		Stroke	Heart disease
N		75,292	68,354		7,790	6,717		13,181	11,834
Incidence rate (%)		3,646 (4.84)	7,552 (11.05)		535 (6.9)	1,073 (16.0)		226 (1.7)	884 (7.5)
Age (years)		63.7 (9.7)	63.1 (9.5)		64.2 (10.2)	63.4 (9.9)		58.7 (9.6)	58.4 (9.6)
Male (%)		34,664 (46.0)	30,662 (44.9)		3,406 (43.7)	2,833 (42.2)		6,295 (47.8)	5,793 (49.0)
Education									
I (%)		31,505 (41.8)	27,933 (40.9)		1,345 (17.3)	1,159 (17.3)		11,774 (89.3)	10,607 (89.6)
II (%)		27,674 (36.8)	25,430 (37.2)		2,697 (34.6)	2,308 (34.4)		1,201 (9.1)	1,053 (8.9)
III (%)		16,113 (21.4)	14,991 (21.9)		3,747 (48.1)	3,249 (48.4)		205 (1.6)	174 (1.5)
BMI (kg/m^2^)		26.9 (4.6)	26.7 (4.5)		29.4 (5.9)	29.4 (5.9)		23.5 (3.9)	23.4 (3.8)
Current smoke (%)		14,579 (19.4)	13,639 (20.0)		1,190 (15.3)	1,088 (16.6)		4,101 (31.1)	3,789 (32.0)
Current drink (%)		36,028 (47.9)	33,238 (48.7)		2,949 (38.5)	2,706 (41.0)		5,246 (39.8)	4,826 (40.8)
Rural (%)		29,528 (39.2)	28,385 (43.2)					8,421 (63.9)	7,703 (65.1)
Dominant HGS (kg)		33.9 (12.3)	34.0 (12.3)		33.6 (11.4)	33.7 (11.5)		30.7 (10.7)	30.9 (10.7)
Relative HGS (m^2^)		2.5 (0.9)	2.5 (0.9)		2.2 (0.8)	2.2 (0.8)		2.7 (1.0)	2.7 (1.0)
Absolute HGS (kg)		65.2 (23.6)	65.4 (23.5)		63.3 (22.2)	63.5 (22.1)		62.0 (21.5)	62.4 (21.1)
Hypertension (%)		27,359 (36.3)	23,656 (34.6)		3,817 (49.8)	3,114 (47.2)		3,310 (25.1)	2,693 (22.8)
Lung problems (%)		4,134 (5.5)	3,327 (4.9)		501 (6.5)	357 (5.4)		1,427 (10.8)	1,157 (9.8)
Cancer (%)		4,443 (5.9)	3,944 (5.8)		884 (11.5)	715 (10.8)		137 (1.04)	109 (0.9)
Diabetes (%)		8,126 (10.8)	6,711 (9.8)		1,278 (16.7)	997 (15.1)		802 (6.1)	632 (5.3)

**Note**: Education I, less than upper secondary; Education II, education/upper secondary; Education III, vocational education/tertiary education; BMI, body mass index; HGS: handgrip strength. Values were presented as n (%) or mean (standard deviation)

After multivariate adjustment, compared with individuals in Q4, the HR values of Q1 of dominant HGS, absolute HGS and relative HGS for stroke were 1.46 (1.30 1.62), 1.63 (1.41–1.89) and 1.56 (1.40–1.75), respectively. With respect to heart disease, the corresponding HRs (95% CIs) were 1.22 (1.13–1.31), 1.33 (1.21–1.47) and 1.41 (1.30–1.52) (Table 2). In addition, for per SD decrease in HGS, the risk of developing stroke and heart disease is 1.21 (1.15–1.27) to 1.27 (1.21–1.33) times and 1.10 (1.06–1.14) to 1.19 (1.15–1.23) times higher. Moreover, after adding dominant HGS, absolute HGS and relative HGS to the office-based risk score, the increment of Harrell’s C index ranged from 0.0047 (95% CI: 0.0030–0.0063) to 0.0058 (95% CI: 0.0039–0.0076) for stroke and 0.0010 (95% CI: 0.0002–0.0017) to 0.0013 (95% CI: 0.0006–0.0021) for heart disease (all *P* < 0.05). However, there were minimal or no significant differences in Harrell’s C index increments among the three HGS expressions (Table 3).

**Table 2:** The associations between baseline HGS and future stroke and heart disease

		SHARE			HRS			CHARLS	
		HR (95% CI)	*P*		HR (95% CI)	*P*		HR (95% CI)	*P*
**Stroke**									
Dominant HGS									
Group 4		Ref	-		Ref	-		Ref	-
Group 3		1.23 (1.11–1.37)	< 0.001		1.22 (0.90–1.64)	0.1944		0.90 (0.58–1.41)	0.6418
Group 2		1.28 (1.15–1.42)	< 0.001		1.64 (1.23–2.17)	0.0006		1.12 (0.73–1.72)	0.6126
Group 1		1.46 (1.30–1.62)	< 0.001		1.71 (1.28–2.28)	0.0003		2.10 (1.39–3.17)	0.0005
Continuous (per SD)		1.21 (1.15–1.27)	< 0.001		1.34 (1.16–1.54)	< 0.001		1.38 (1.16–1.63)	0.0002
Relative HGS									
Group 4		Ref	-		Ref	-		Ref	-
Group 3		1.16 (1.04–1.30)	0.0104		1.32 (0.98–1.78)	0.0685		1.24 (0.77-2.00)	0.3808
Group 2		1.33 (1.19–1.49)	< 0.001		1.50 (1.12–2.01)	0.0064		1.87 (1.20–2.91)	0.0057
Group 1		1.56 (1.40–1.75)	< 0.001		1.70 (1.27–2.28)	0.0004		2.07 (1.33–3.23)	0.0014
Continuous (per SD)		1.27 (1.21–1.33)	< 0.001		1.26 (1.10–1.44)	0.0008		1.42 (1.18–1.71)	0.0002
Absolute HGS									
Group 4		Ref	-		Ref	-		Ref	-
Group 3		1.22 (1.04–1.42)	0.0134		1.33 (0.98–1.81)	0.0688		1.02 (0.66–1.58)	0.9361
Group 2		1.28 (1.11–1.47)	0.0006		1.57 (1.17–2.12)	0.0026		1.16 (0.76–1.77)	0.4955
Group 1		1.63 (1.41–1.89)	< 0.001		1.83 (1.36–2.47)	< 0.001		1.78 (1.17–2.70)	0.0073
Continuous (per SD)		1.25 (1.18–1.31)	< 0.001		1.34 (1.16–1.54)	< 0.001		1.32 (1.10–1.59)	0.0033
**Heart disease**									
Dominant HGS									
Group 4		Ref	-		Ref	-		Ref	-
Group 3		1.07 (1.00-1.15)	0.0699		1.32 (1.09–1.59)	0.0046		0.86 (0.71–1.04)	0.1266
Group 2		1.09 (1.02–1.17)	0.0140		1.16 (0.95–1.40)	0.1395		0.88 (0.72–1.07)	0.2055
Group 1		1.22 (1.13–1.31)	< 0.001		1.32 (1.08–1.62)	0.0060		0.91 (0.74–1.12)	0.3662
Continuous (per SD)		1.10 (1.06–1.14)	< 0.001		1.15 (1.04–1.27)	0.0089		0.99 (0.90–1.09)	0.8187
Relative HGS									
Group 4		Ref	-		Ref	-		Ref	-
Group 3		1.13 (1.04–1.22)	0.0021		0.97 (0.80–1.18)	0.7811		0.96 (0.78–1.18)	0.6864
Group 2		1.24 (1.15–1.34)	< 0.001		1.08 (0.90–1.30)	0.4178		1.13 (0.93–1.38)	0.2338
Group 1		1.41 (1.30–1.52)	< 0.001		1.26 (1.04–1.52)	0.0167		1.13 (0.92–1.38)	0.2440
Continuous (per SD)		1.19 (1.15–1.23)	< 0.001		1.13 (1.03–1.24)	0.0103		1.05 (0.96–1.15)	0.3254
Absolute HGS									
Group 4		Ref	-		Ref	-		Ref	-
Group 3		1.17 (1.06–1.28)	0.0017		1.18 (0.97–1.42)	0.0928		0.88 (0.72–1.07)	0.1963
Group 2		1.14 (1.04–1.26)	0.0070		1.07 (0.89–1.30)	0.4750		0.95 (0.78–1.15)	0.5704
Group 1		1.33 (1.21–1.47)	< 0.001		1.26 (1.03–1.53)	0.0245		0.89 (0.72–1.10)	0.2780
Continuous (per SD)		1.11 (1.07–1.15)	< 0.001		1.12 (1.01–1.24)	0.0320		0.97 (0.89–1.07)	0.5703

**Note**: Group 1 = the first quartile (lowest); Group 2 = the second quartile; Group 3 = the third quartile; Group 4 = Reference (Ref) = the fourth quartile; Dominant HGS: maximum HGS of dominant hand; Absolute HGS: the sum of the maximum HGS of both hands; Relative HGS: absolute HGS divided by BMI. Models were adjusted for age, gender, education, residence, BMI (not for relative HGS), smoking status, drinking status, and chronic diseases

**Table 3:** Harrell’s C index changes for stroke and heart disease by adding HGS to office based risk factors

	Harrell’s C index (95% CI)	Harrell’s C index change (95% CI)	*P value*
**Stroke**			
** SHARE**			
Office-based risk factors	0.7081 (0.6993–0.7168)		
Plus dominant HGS	0.7128 (0.7041–0.7214)	0.0047 (0.0030–0.0063)^1^	< 0.0001
Plus relative HGS	0.7136 (0.7049–0.7222)	0.0055 (0.0037–0.0073)^2^	< 0.0001
Plus absolute HGS	0.7139 (0.7052–0.7225)	0.0058 (0.0039–0.0076)^3^	< 0.0001
		0.0008 (0.0000-0.0015)^4^	0.0310^*^
		0.0011 (0.0005–0.0017)^5^	0.0001^*^
		0.0003 (-0.0002-0.0008)^6^	0.2393^*^
** HRS**			
Office-based risk factors	0.7047 (0.6837–0.7257)		
Plus dominant HGS	0.7116 (0.6907–0.7324)	0.0069 (0.0015–0.0122)^1^	0.0116
Plus relative HGS	0.7104 (0.6893–0.7314)	0.0057 (0.0005–0.0108)^2^	0.0306
Plus absolute HGS	0.7109 (0.6900-0.7318)	0.0062 (0.0009–0.0115)^3^	0.0216
		-0.0012 (-0.0035-0.0001)^4^	0.2971^*^
		-0.0006 (-0.0019-0.0007)^5^	0.3392^*^
		0.0006 (-0.0013-0.0024)^6^	0.5490^*^
** CHARLS**			
Office-based risk factors	0.6697 (0.6342–0.7052)		
Plus dominant HGS	0.6815 (0.6464–0.7167)	0.0120 (-0.0020-0.0260)^1^	0.0921
Plus relative HGS	0.6808 (0.6460–0.7155)	0.0111 (-0.0029-0.0250)^2^	0.1196
Plus absolute HGS	0.6771 (0.6418–0.7123)	0.0074 (-0.0047-0.0195)^3^	0.2352
		-0.0009 (-0.0072-0.0053)^4^	0.7669^*^
		-0.0046 (-0.0098-0.0006)^5^	0.0807^*^
		-0.0037 (-0.0077-0.0003)^6^	0.0700^*^
**Heart disease**			
** SHARE**			
Office-based risk factors	0.6915 (0.6854–0.6977)		
Plus dominant HGS	0.6927 (0.6865–0.6988)	0.0011 (0.0004–0.0018)^1^	0.0021
Plus relative HGS	0.6925 (0.6864–0.6986)	0.0010 (0.0002–0.0017)^2^	0.0103
Plus absolute HGS	0.6929 (0.6868–0.6990)	0.0013 (0.0006–0.0021)^3^	0.0005
		-0.0001 (-0.0004-0.0001)^4^	0.3266^*^
		0.0002 (0.0000-0.0005)^5^	0.0540^*^
		0.0004 (0.0002–0.0006)^6^	0.0003^*^
** HRS**			
Office-based risk factors	0.6558 (0.6394–0.6721)		
Plus dominant HGS	0.6590 (0.6427–0.6752)	0.0033 (0.0004–0.0061)^1^	0.0244
Plus relative HGS	0.6575 (0.6412–0.6738)	0.0018 (-0.0002-0.0037)^2^	0.0760
Plus absolute HGS	0.6581 (0.6418–0.6744)	0.0023 (-0.0001-0.0047)^3^	0.0615
		-0.0015 (-0.0029–0.0001)^4^	0.0317^*^
		-0.0010 (-0.0018–0.0002)^5^	0.0192^*^
		0.0005 (-0.0004-0.0014)^6^	0.2415^*^
** CHARLS**			
Office-based risk factors	0.6429 (0.6244–0.6614)		
Plus dominant HGS	0.6428 (0.6242–0.6613)	-0.0002 (-0.0013-0.0009)^1^	0.7763
Plus relative HGS	0.6429 (0.6244–0.6615)	0.0000 (-0.0010-0.0011)^2^	0.9483
Plus absolute HGS	0.6427 (0.6242–0.6613)	-0.0002 (-0.0016-0.0013)^3^	0.8117
		0.0002 (-0.0003-0.0007)^4^	0.4670^*^
		0.0000 (-0.0007-0.0006)^5^	0.9516^*^
		-0.0002 (-0.0007-0.0003)^6^	0.4276^*^

**Note**: HGS: handgrip strength

^1^ Harrell’s C index change between dominant HGS + office based risk factors (age, gender, BMI, hypertension, smoking and diabetes) and office based risk factors;

^2^ Harrell’s C index change between relative HGS + office based risk factors and office based risk factors;

^3^ Harrell’s C index change between absolute HGS + office based risk factors and office based risk factors;

^4^ Harrell’s C index difference between dominant HGS + office based risk factors and relative HGS + office based risk factors;

^5^ Harrell’s C index difference between dominant HGS + office based risk factors and absolute HGS + office based risk factors;

^6^ Harrell’s C index difference between relative HGS + office based risk factors and absolute HGS + office based risk factors;

*Analyses involving comparisons of three HGS forms with each other were adjusted using the Bonferroni adjustment method, and *P* < 0.05/3 was considered as statistically significant.

## Results from HRS

Based on the inclusion and exclusion criteria, 7,790 participants (mean age of 64.2 years, 56.3% women) in the “stroke” study and 6,717 (mean age of 63.4 years, 57.8% women) in the “heart disease” study were included. With a median follow-up time of 8 and 7.04 years, 535 participants suffered from stroke and 1,073 from heart disease (Table 1).

Table 2 summarizes the results of the fully adjusted model. Participants in Q1 of HGS also possessed higher risk of stroke than those in Q4, with HRs (95% CIs) being 1.71 (1.28–2.28) of dominant HGS, 1.83 (1.36–2.47) of absolute HGS and 1.70 (1.27–2.28) of relative HGS, respectively. For heart disease, they were 1.32 (1.08–1.62), 1.26 (1.03–1.53) and 1.26 (1.04–1.52), respectively. In addition, for per SD decrease in HGS, risks of developing stroke and heart disease were 1.26 (1.10–1.44) to 1.34 (1.16–1.54) times and 1.12 (1.01–1.24) to 1.15 (1.04–1.27) times higher (Table 2). Adding HGS to office-based factors significantly improved the predictive abilities of stroke and heart disease. However, there were subtle differences or no significant differences between the Harrell’s C index increments of any two HGS forms (all *P* > 0.05/3) (Table 3).

## Results from CHARLS

A total of 13,181 individuals (mean age of 58.7 years, 52.2% women) in the “stroke” study and 11,834 individuals (mean age of 58.4 years, 51.0% women) in the “heart disease” study was ultimately included. With a median follow-up time of 4 years, 226 participants suffered from stroke and 884 from heart disease (Table 1).

After adjusting for sociodemographic factors, lifestyle factors and common chronic diseases, we observed a positive association of low HGS with stroke but not with heart disease. Compared to the highest quartile, the HR (95% CI) values of the Q1 of dominant HGS, absolute HGS and relative HGS for stroke were 2.10 (1.39–3.17), 1.78 (1.17–2.70), and 2.07 (1.33–3.23), respectively (Table 2). When the three HGS forms were added to the office-based risk factors for predicting stroke, the increments in Harrell’s C index were 0.0120 (95% CI: -0.0020-0.0260) for dominant HGS, 0.0111 (95% CI: -0.0029-0.0250) for relative HGS and 0.0074 (95% CI: -0.0047-0.0195) for absolute HGS, respectively, again not statistically different (Table 3).

## Subgroup and sensitivity analysis

In stratified analyses by gender and age (< 60 or ≥ 60 years), HGS showed a stable association with the risk of stroke in SHARE, HRS and CHARLS, especially in women. However, the significant association between HGS and heart disease was only observed in the subgroups in SHARE and the subgroup of age under the 60 in HRS, but not in CHARLS. Moreover, the Fine-Gray competitive risk model showed a similar result pattern to the main analyses. The corresponding HRs (95% CI) were presented in Supplemental Tables 2–4.

## Discussion

Consistent results from three prospective cohorts showed that lower HGS was associated with a higher risk of stroke. In addition, dominant HGS, absolute HGS and relative HGS contributed to improve the predictive ability of stroke, but there were minimal or no differences in the increases of Harrell’s C indexes among three HGS expressions. However, this phenomenon related to heart disease was only observed in the middle-aged and older European and American populations.

Many previous studies have reported a correlation between baseline HGS and the risk of cardiovascular disease [11, 29, 30]. Kasper Andersen et al. suggested that HGS was closely related with risks of stroke and ischemic heart disease among young Swedish men [29]. Likewise, a Mendelian randomization study reported that higher HGS reduced the risk of CHD with a 1-kilogram increase [30]. Moreover, the PURE study also demonstrated that HGS measurement, rather than systolic blood pressure, was a simpler and inexpensive way to stratify risks of stroke and heart disease in 17 countries [11]. However, there were several inconsistent findings. For example, a CoLaus population-based study demonstrated that there was no significant association between HGS and cardiovascular risk after adjusting for baseline covariates [14]. Nevertheless, this study only included 2,707 participants from Switzerland, which may result in insufficient power. In addition, Fujita et al. also suggested that the correlation between HGS and cardiovascular disease risk disappeared after adjusting for age, blood pressure, smoking status, and other factors among Japanese individuals aged over 40 [31]. Furthermore, although the association between HGS and the risk of stroke has been reported in Chinese populations, our study had a broader population base and added a comparison of different HGS expressions [32]. This study, which took into account the results from three nationally representative cohorts, supported a significant negative association between HGS and the risk of stroke.

Moreover, the Korean Longitudinal Study of Aging (KLOSA) showed that the predictive value of relative HGS was superior to dominant HGS, by comparing the Quasi-Akaike Information Criterion values of dominant HGS, absolute HGS and relative HGS [15]. Similarly, Hannah G et al. suggested that relative HGS might be a better health biomarker among U.S. adults as it was associated with a broader range of cardiovascular markers than absolute HGS [33]. The potential explanations might be that relative HGS (absolute strength corrected for a measure of body size such as BMI) address both the confounding of strength by body mass and concomitant health risks of increased body weight and low muscular strength [33]. However, Frederick K et al. suggested that the ability of HGS to predict cardiovascular mortality was independent of how it was expressed based on results from the UK Biobank [28]. Our study also showed that the abilities of HGS to predict new-onset stroke did not differ regardless of whether HGS was expressed in absolute or relative terms.

The modest association between HGS and heart disease was only seen in SHARE and HRS, but not in CHARLS. We attempted to explain this phenomenon in two ways. First, there are differences in the physical conditions of European, Chinese, and American, such as HGS, BMI, and waist circumference [34]. Inherent differences in body composition of different ethnic groups may affect the final results. In addition, the definition of heart disease in the three studies was broad and not specific to CHD, heart failure (HF), or myocardial infarction (MI). Future studies confined to the specific endpoints of CHD, HF, or MI need to be conducted and compared.

The underlying mechanisms by which HGS is associated with cardiovascular disease have not been fully elaborated. Previous studies have suggested that HGS is closely related to cardiometabolic risk factors, such as glycohemoglobin (HbA1c) and uric acid (UA) [35, 36]. In addition to assisting in the diagnosis of diabetes, HbA1c has also been applied to explain the occurrence of cardiovascular diseases such as coronary heart disease and ischemic stroke in many fields [24]. Many epidemiological studies have also shown the association between elevated serum UA levels and CVD, including CHD, stroke, and other diseases. The intermediate processes may include excessive UA causing increased oxidative stress, decreased nitric oxide availability, endothelial dysfunction, promotion of inflammation, vasoconstriction, and vascular smooth muscle cell proliferation [37, 38]. In addition, HGS has been considered a well-established indicator of muscle strength, which is associated with the basal metabolic rate and other metabolic features [15, 39]. The body’s metabolism slows down when there is less muscle, and various metabolic diseases may occur [39]. Moreover, it has been reported that age-related vascular dysfunction is an important contributory factor to anabolic resistance, which is the primary driver of sarcopenia in ageing populations [40].

This study included three cohorts representing Asian, European, and American populations to comprehensively investigate the associations between HGS and stroke and heart disease. In addition, we further verified whether the predictive values of HGS in stroke and heart diseases depended on how it was expressed. However, some limitations of our study are still worth noting. First, given a limited number of covariates considered in the statistical model and the self-reported nature of the potential confounders, residual confounding from unmeasured or inaccurate confounders is likely. For example, measures of physical activity were not standardized across these three different cohorts, so we did not include it in the adjustment model. Second, because of the limitation of data availability, the definitions of chronic diseases were determined using self-reported information. However, self-reported results have been shown to remain reliable in large-scale epidemiological surveys [41]. Third, heart disease is a broad term that includes myocardial infarction, coronary thrombosis and any other heart problem in this study, and future studies could limit the outcome to specific heart symptoms. Finally, due to the self-reported nature of outcomes, only non-fatal stroke and heart disease events were included in our study. Further studies are warranted to explore the associations between HGS and fatal events.

## Conclusion

This prospective study showed that HGS could be used as an independent predictor of stroke in middle-aged and older European, American and Chinese populations, and the predictive ability of HGS might not depend on how it is expressed. However, the modest association between HGS and heart disease was only observed in the middle-aged and older European and American populations, which calls for further validation. Our study highlights that increased attention should be given to middle-aged and older people with low HGS to prevent the development of stroke and heart disease in the future.

## Electronic supplementary material

Below is the link to the electronic supplementary material.

**Supplementary Table 1**. The ranges for gender-specific quartiles of HGS in the 3 cohorts.
**Supplementary Table 2**. Subgroup analysis by gender for the associations between baseline HGS and future stroke and heart disease.
**Supplementary Table 3**. Subgroup analysis by age for the associations between baseline HGS and future stroke and heart disease.
**Supplementary Table 4**. Associations between baseline HGS and future stroke and heart disease using competing risk model.

## Data Availability

This analysis uses data or information from the Harmonized SHARE dataset and Codebook, Version E as of October 2019, the Harmonized HRS dataset and Codebook, RAND HRS Longitudinal File 2016 (V2) Documentation as well as the Harmonized CHARLS dataset and Codebook, Version C as of April 2018. All information was developed by the Gateway to Global Aging Data. The development of the Harmonized SHARE was funded by the National Institute on Ageing (R01 AG030153, RC2 AG036619, R03 AG043052), HRS was funded by the Social Security Administration and the National Institute on Aging Produced by the RAND Center for the Study of Aging, and the development of the Harmonized CHARLS was funded by the National Institute on Ageing (R01 AG030153, RC2 AG036619, R03 AG043052). The data used from the 3 prospective cohorts in the study is open access/publicly available. If you would like to obtain data for this study, please refer to www.g2aging.org. or contact the corresponding author.

## List of Abbreviations

HGS: handgrip strength
CVD: cardiovascular disease
CHD: coronary heart disease
PURE: the Prospective Urban Rural Epidemiology study
SHARE: the Survey of Health Ageing and Retirement in Europe
HRS: the Health and Retirement Study
CHARLS: the China Health and Retirement Longitudinal Study
BMI: body mass index
KLOSA: the Korean Longitudinal Study of Aging
UA: uric acid
HF: heart failure
MI: myocardial infarction
