# Supplementary material for: Handgrip strength is associated with risks of new-onset stroke and heart disease: results from 3 prospective cohorts
Source: BMC Geriatr. 2023 May 4;23:268. doi: 10.1186/s12877-023-03953-8 (PMC10161641; doi:10.1186/s12877-023-03953-8)
Supplement: Supplementary file 1 — Supplementary Table 1. The ranges for gender-specific quartiles of HGS in the 3 cohorts. Supplementary Table 2. Subgroup analysis by gender for the associations between baseline HGS and future stroke and heart disease. Supplementary Table 3. Subgroup analysis by age for the associations between baseline HGS and future stroke and heart disease. Supplementary Table 4. Associations between baseline HGS and future stroke and heart disease using competing risk model. [file 12877_2023_3953_MOESM1_ESM.docx]

**Supplementary materials**

**Handgrip strength is associated with risks of new-onset stroke and heart disease: results from 3 prospective cohorts**

**Supplementary Table 1** The ranges for gender-specific quartiles of HGS in the 3 cohorts

|  |  | **SHARE** | |  | **HRS** | |  | **CHARLS** | |
| --- | --- | --- | --- | --- | --- | --- | --- | --- | --- |
|  |  | Man | Women |  | Man | Women |  | Man | Women |
| **Stroke** |  |  |  |  |  |  |  |  |  |
| Dominant HGS |  |  |  |  |  |  |  |  |  |
| Group 4 |  | ≥50 | ≥31 |  | ≥49.5 | ≥30 |  | ≥43.75 | ≥29.75 |
| Group 3 |  | 43~50 | 26~31 |  | 43~49.5 | 26~30 |  | 37.5~43.75 | 25~29.75 |
| Group 2 |  | 36~43 | 22~26 |  | 37~43 | 22~26 |  | 31~37.5 | 20.25~25 |
| Group 1 |  | ≤36 | ≤22 |  | ≤37 | ≤22 |  | ≤31 | ≤20.25 |
| Relative HGS |  |  |  |  |  |  |  |  |  |
| Group 4 |  | ≥3.65 | ≥2.35 |  | ≥3.33 | ≥2.04 |  | ≥3.83 | ≥2.55 |
| Group 3 |  | 3.10~3.65 | 1.92~2.35 |  | 2.82~3.33 | 1.68~2.04 |  | 3.33~3.83 | 2.14~2.55 |
| Group 2 |  | 2.56~3.10 | 1.52~1.92 |  | 2.34~2.82 | 1.37~1.68 |  | 2.78~3.33 | 1.72~2.14 |
| Group 1 |  | ≤2.56 | ≤1.52 |  | ≤2.34 | ≤1.37 |  | ≤2.78 | ≤1.72 |
| Absolute HGS |  |  |  |  |  |  |  |  |  |
| Group 4 |  | ≥97 | ≥59 |  | ≥94.5 | ≥57 |  | ≥87 | ≥60 |
| Group 3 |  | 84~97 | 55~59 |  | 81.5~94.5 | 49.5~57 |  | 75.7~87 | 51~60 |
| Group 2 |  | 70~84 | 41~55 |  | 69.5~81.5 | 41~49.5 |  | 63~75.7 | 41~51 |
| Group 1 |  | ≤70 | ≤41 |  | ≤69.5 | ≤41 |  | ≤63 | ≤41 |
| **Heart disease** |  |  |  |  |  |  |  |  |  |
| Dominant HGS |  |  |  |  |  |  |  |  |  |
| Group 4 |  | ≥50 | ≥31 |  | ≥50 | ≥30.5 |  | ≥43.5 | ≥29.55 |
| Group 3 |  | 44~50 | 27~31 |  | 44~50 | 26.5~30.5 |  | 37.5~43.5 | 25~29.55 |
| Group 2 |  | 37~44 | 22~27 |  | 37~44 | 22~26.5 |  | 31~37.5 | 20~25 |
| Group 1 |  | ≤37 | ≤22 |  | ≤37 | ≤22 |  | ≤31 | ≤20 |
| Relative HGS |  |  |  |  |  |  |  |  |  |
| Group 4 |  | ≥3.69 | ≥2.37 |  | ≥3.38 | ≥2.06 |  | ≥3.82 | ≥2.53 |
| Group 3 |  | 3.14~3.69 | 1.95~2.37 |  | 2.85~3.38 | 1.71~2.06 |  | 3.31~3.82 | 2.12~2.53 |
| Group 2 |  | 2.60~3.14 | 1.55~1.95 |  | 2.39~2.85 | 1.39~1.71 |  | 2.77~3.31 | 1.72~2.12 |
| Group 1 |  | ≤2.60 | ≤1.55 |  | ≤2.39 | ≤1.39 |  | ≤2.77 | ≤1.72 |
| Absolute HGS |  |  |  |  |  |  |  |  |  |
| Group 4 |  | ≥98 | ≥60 |  | ≥95 | ≥57.5 |  | ≥87 | ≥60 |
| Group 3 |  | 85~98 | 51~60 |  | 82.5~95 | 50~57.5 |  | 75.5~87 | 50.5~60 |
| Group 2 |  | 71~85 | 42~51 |  | 70~82.5 | 41~50 |  | 63~75.5 | 41~50.5 |
| Group 1 |  | ≤71 | ≤42 |  | ≤70 | ≤41 |  | ≤63 | ≤41 |

**Note:** Group 1= the first quartile (lowest); Group 2=the second quartile; Group 3= the third quartile; Group 4= the fourth quartile; Dominant HGS: maximum HGS of dominant hand; Absolute HGS: the sum of the maximum HGS of both hands; Relative HGS: absolute HGS divided by BMI.

**Supplementary Table 2** Subgroup analysis by gender for the associations between baseline HGS and future stroke and heart disease

|  |  |  | SHARE | |  | HRS | |  | CHARLS | |
| --- | --- | --- | --- | --- | --- | --- | --- | --- | --- | --- |
|  |  |  | Men | Women |  | Men | Women |  | Men | Women |
| **Stroke** | |  |  |  |  |  |  |  |  |  |
|  | Dominant HGS |  |  |  |  |  |  |  |  |  |
|  |  | Group 4 | 1 (Ref) | 1 (Ref) |  | 1 (Ref) | 1 (Ref) |  | 1 (Ref) | 1 (Ref) |
|  |  | Group 3 | 1.31 (1.14-1.52) | 1.15 (0.98-1.35) |  | 1.47 (0.93-2.34) | 1.05 (0.71-1.56) |  | 0.88 (0.50-1.55) | 0.95 (0.46-1.97) |
|  |  | Group 2 | 1.28 (1.10-1.48) | 1.30 (1.10-1.52) |  | 2.06 (1.32-3.20) | 1.37 (0.94-1.98) |  | 1.10 (0.63-1.91) | 1.17 (0.58-2.34) |
|  |  | Group 1 | 1.48 (1.27-1.72) | 1.45 (1.24-1.70) |  | 1.70 (1.07-2.71) | 1.68 (1.17-2.43) |  | 1.57 (0.90-2.73) | 2.93 (1.54-5.57) |
|  |  | Continuous (per SD) | 1.16 (1.10-1.22) | 1.18 (1.12-1.25) |  | 1.21 (1.04-1.41) | 1.26 (1.11-1.44) |  | 1.23 (1.01-1.49) | 1.45 (1.18-1.78) |
|  | Relative HGS |  |  |  |  |  |  |  |  |  |
|  |  | Group 4 | 1 (Ref) | 1 (Ref) |  | 1 (Ref) | 1 (Ref) |  | 1 (Ref) | 1 (Ref) |
|  |  | Group 3 | 1.15 (0.98-1.34) | 1.19 (1.00-1.41) |  | 1.35 (0.87-2.10) | 1.31 (0.87-1.96) |  | 0.87 (0.46-1.64) | 2.03 (0.96-4.33) |
|  |  | Group 2 | 1.35 (1.16-1.57) | 1.32 (1.12-1.56) |  | 1.50 (0.97-2.30) | 1.47 (0.99-2.18) |  | 1.78 (1.02-3.09) | 1.95 (0.92-4.12) |
|  |  | Group 1 | 1.53 (1.31-1.78) | 1.63 (1.38-1.93) |  | 1.56 (1.00-2.42) | 1.77 (1.19-2.63) |  | 1.43 (0.80-2.57) | 3.21 (1.58-6.55) |
|  |  | Continuous (per SD) | 1.27 (1.21-1.33) | 1.27 (1.21-1.33) |  | 1.19 (1.02-1.38) | 1.20 (1.04-1.38) |  | 1.26 (1.03-1.56) | 1.42 (1.13-1.78) |
|  | Absolute HGS |  |  |  |  |  |  |  |  |  |
|  |  | Group 4 | 1 (Ref) | 1 (Ref) |  | 1 (Ref) | 1 (Ref) |  | 1 (Ref) | 1 (Ref) |
|  |  | Group 3 | 1.21 (1.04-1.40) | 1.00 (0.81-1.23) |  | 1.28 (0.82-2.02) | 1.38 (0.91-2.09) |  | 0.92 (0.54-1.58) | 1.34 (0.62-2.90) |
|  |  | Group 2 | 1.20 (1.03-1.39) | 1.24 (1.07-1.43) |  | 1.70 (1.10-2.63) | 1.46 (0.98-2.19) |  | 0.84 (0.49-1.45) | 1.99 (0.97-4.08) |
|  |  | Group 1 | 1.53 (1.30-1.78) | 1.55 (1.32-1.82) |  | 1.47 (0.93-2.33) | 2.10 (1.41-3.13) |  | 1.21 (0.70-2.11) | 3.09 (1.53-6.23) |
|  |  | Continuous (per SD) | 1.19 (1.12-1.25) | 1.21 (1.14-1.28) |  | 1.18 (1.02-1.38) | 1.29 (1.13-1.47) |  | 1.18 (0.95-1.46) | 1.43 (1.14-1.78) |
| **Heart diseases** | |  |  |  |  |  |  |  |  |  |
|  | Dominant HGS |  |  |  |  |  |  |  |  |  |
|  |  | Group 4 | 1 (Ref) | 1 (Ref) |  | 1 (Ref) | 1 (Ref) |  | 1 (Ref) | 1 (Ref) |
|  |  | Group 3 | 1.07 (0.97-1.18) | 1.08 (0.97-1.20) |  | 1.33 (1.00-1.76) | 1.31 (1.01-1.69) |  | 0.92 (0.68-1.23) | 0.82 (0.63-1.06) |
|  |  | Group 2 | 1.05 (0.96-1.16) | 1.14 (1.03-1.26) |  | 1.06 (0.80-1.42) | 1.23 (0.95-1.59) |  | 0.71 (0.51-0.98) | 1.00 (0.78-1.29) |
|  |  | Group 1 | 1.18 (1.07-1.31) | 1.28 (1.15-1.42) |  | 1.32 (0.99-1.78) | 1.34 (1.02-1.76) |  | 0.80 (0.57-1.12) | 0.96 (0.74-1.25) |
|  |  | Continuous (per SD) | 1.08 (1.04-1.13) | 1.09 (1.05-1.13) |  | 1.11 (1.00-1.24) | 1.09 (0.99-1.20) |  | 0.95 (0.84-1.07) | 1.02 (0.92-1.12) |
|  | Relative HGS |  |  |  |  |  |  |  |  |  |
|  |  | Group 4 | 1 (Ref) | 1 (Ref) |  | 1 (Ref) | 1 (Ref) |  | 1 (Ref) | 1 (Ref) |
|  |  | Group 3 | 1.09 (0.98-1.20) | 1.19 (1.06-1.34) |  | 0.89 (0.66-1.18) | 1.05 (0.81-1.36) |  | 0.98 (0.71-1.35) | 0.95 (0.72-1.25) |
|  |  | Group 2 | 1.15 (1.04-1.28) | 1.38 (1.23-1.54) |  | 1.03 (0.78-1.36) | 1.13 (0.88-1.46) |  | 0.98 (0.72-1.35) | 1.21 (0.94-1.57) |
|  |  | Group 1 | 1.29 (1.16-1.44) | 1.58 (1.41-1.77) |  | 1.20 (0.91-1.58) | 1.31 (1.02-1.70) |  | 0.98 (0.70-1.35) | 1.23 (0.95-1.59) |
|  |  | Continuous (per SD) | 1.13 (1.09-1.17) | 1.21 (1.17-1.26) |  | 1.08 (0.97-1.20) | 1.12 (1.02-1.23) |  | 0.99 (0.88-1.11) | 1.07 (0.97-1.18) |
|  | Absolute HGS |  |  |  |  |  |  |  |  |  |
|  |  | Group 4 | 1 (Ref) | 1 (Ref) |  | 1 (Ref) | 1 (Ref) |  | 1 (Ref) | 1 (Ref) |
|  |  | Group 3 | 1.11 (1.00-1.23) | 1.08 (0.97-1.20) |  | 1.19 (0.90-1.58) | 1.17 (0.90-1.51) |  | 0.864 (0.64-1.17) | 0.89 (0.69-1.16) |
|  |  | Group 2 | 1.04 (0.94-1.15) | 1.10 (0.99-1.23) |  | 1.00 (0.74-1.33) | 1.14 (0.89-1.47) |  | 0.82 (0.60-1.11) | 1.03 (0.80-1.33) |
|  |  | Group 1 | 1.23 (1.11-1.37) | 1.28 (1.15-1.42) |  | 1.26 (0.93-1.69) | 1.26 (0.96-1.64) |  | 0.71 (0.51-0.99) | 1.01 (0.77-1.32) |
|  |  | Continuous (per SD) | 1.09 (1.05-1.13) | 1.70 (1.06-1.14) |  | 1.09 (0.98-1.21) | 1.08 (0.98-1.19) |  | 0.92 (0.82-1.04) | 1.01 (0.92-1.11) |

**Note:** Group 1= the first quartile (lowest); Group 2=the second quartile; Group 3= the third quartile; Group 4= reference=the fourth quartile; Dominant HGS: maximum HGS of dominant hand; Absolute HGS: the sum of the maximum HGS of both hands; Relative HGS: absolute HGS divided by BMI.

**Supplementary Table 3** Subgroup analysis by age for the associations between baseline HGS and future stroke and heart disease

|  |  |  | SHARE | |  | HRS | |  | CHARLS | |
| --- | --- | --- | --- | --- | --- | --- | --- | --- | --- | --- |
|  |  |  | Age< 60 | Age≥ 60 |  | Age< 60 | Age≥ 60 |  | Age< 60 | Age≥ 60 |
| **Stroke** | |  |  |  |  |  |  |  |  |  |
|  | Dominant HGS |  |  |  |  |  |  |  |  |  |
|  |  | Group 4 | 1 (Ref) | 1 (Ref) |  | 1 (Ref) | 1 (Ref) |  | 1 (Ref) | 1 (Ref) |
|  |  | Group 3 | 1.27 (1.04-1.56) | 1.09 (0.98-1.22) |  | 1.24 (0.67-2.30) | 1.07 (0.78-1.46) |  | 0.84 (0.46-1.53) | 1.60 (0.93-2.75) |
|  |  | Group 2 | 1.63 (1.23-2.14) | 1.18 (1.03-1.35) |  | 2.21 (1.06-4.61) | 1.05 (0.70-1.58) |  | 1.14 (0.56-2.29) | 1.98 (1.09-3.58) |
|  |  | Group 1 | 2.20 (1.66-2.91) | 1.49 (1.28-1.72) |  | 3.00 (1.39-6.45) | 1.61 (1.05-2.46) |  | 1.80 (0.87-3.73) | 3.60 (1.94-6.65) |
|  |  | Continuous (per SD) | 1.36 (1.23-1.50) | 1.16 (1.10-1.23) |  | 1.45 (1.09-1.94) | 1.22 (1.05-1.41) |  | 1.19 (0.92-1.55) | 1.50 (1.22-1.84) |
|  | Relative HGS |  |  |  |  |  |  |  |  |  |
|  |  | Group 4 | 1 (Ref) | 1 (Ref) |  | 1 (Ref) | 1 (Ref) |  | 1 (Ref) | 1 (Ref) |
|  |  | Group 3 | 1.15 (0.933-1.42) | 1.11 (0.99-1.24) |  | 0.57 (0.29-1.12) | 1.18 (0.87-1.60) |  | 1.24 (0.69-2.24) | 1.40 (0.81-2.43) |
|  |  | Group 2 | 1.31 (1.02-1.68) | 1.32 (1.16-1.49) |  | 1.39 (0.73-2.66) | 1.14 (0.79-1.63) |  | 1.44 (0.71-2.89) | 1.83 (1.01-3.31) |
|  |  | Group 1 | 1.70 (1.30-2.23) | 1.68 (1.46-1.94) |  | 1.59 (0.77-3.31) | 1.56 (1.06-2.29) |  | 1.44 (0.67-3.10) | 3.10 (1.69-5.68) |
|  |  | Continuous (per SD) | 1.28 (1.16-1.42) | 1.25 (1.18-1.32) |  | 1.27 (0.96-1.68) | 1.26 (1.07-1.47) |  | 1.31 (0.99-1.74) | 1.47 (1.17-1.85) |
|  | Absolute HGS |  |  |  |  |  |  |  |  |  |
|  |  | Group 4 | 1 (Ref) | 1 (Ref) |  | 1 (Ref) | 1 (Ref) |  | 1 (Ref) | 1 (Ref) |
|  |  | Group 3 | 1.23 (1.01-1.51) | 1.10 (0.99-1.23) |  | 1.11 (0.60-2.05) | 0.98 (0.72-1.34) |  | 0.70 (0.38-1.28) | 1.59 (0.93-2.70) |
|  |  | Group 2 | 1.68 (1.28-2.21) | 1.35 (1.18-1.54) |  | 1.86 (0.90-3.84) | 0.97 (0.65-1.45) |  | 0.77 (0.37-1.61) | 1.68 (0.92-3.07) |
|  |  | Group 1 | 2.12 (1.58-2.82) | 1.62 (1.40-1.88) |  | 2.35 (1.11-4.98) | 1.51 (0.98-2.31) |  | 1.34 (0.64-2.79) | 3.04 (1.66-5.57) |
|  |  | Continuous (per SD) | 1.37 (1.23-1.52) | 1.21 (1.14-1.28) |  | 1.40 (1.05-1.86) | 1.27 (1.08-1.490 |  | 1.15 (0.87-1.53) | 1.43 (1.14-1.79) |
| **Heart diseases** | |  |  |  |  |  |  |  |  |  |
|  | Dominant HGS |  |  |  |  |  |  |  |  |  |
|  |  | Group 4 | 1 (Ref) | 1 (Ref) |  | 1 (Ref) | 1 (Ref) |  | 1 (Ref) | 1 (Ref) |
|  |  | Group 3 | 1.06 (0.98-1.14) | 1.10 (0.97-1.25) |  | 0.95 (0.65-1.37) | 1.14 (0.91-1.42) |  | 0.71 (0.52-0.98) | 0.89 (0.67-1.18) |
|  |  | Group 2 | 1.16 (1.05-1.28) | 1.12 (0.93-1.34) |  | 1.19 (0.72-1.95) | 1.16 (0.87-1.54) |  | 0.80 (0.57-1.13) | 0.91 (0.66-1.24) |
|  |  | Group 1 | 1.35 (1.21-1.50) | 1.38 (1.14-1.67) |  | 1.65 (1.00-2.75) | 1.15 (0.84-1.58) |  | 0.94 (0.65-1.35) | 0.86 (0.61-1.21) |
|  |  | Continuous (per SD) | 1.11 (1.07-1.16) | 1.10 (1.03-1.18) |  | 1.23 (1.02-1.48) | 1.09 (0.96-1.23) |  | 1.01 (0.88-1.15) | 0.97 (0.85-1.10) |
|  | Relative HGS |  |  |  |  |  |  |  |  |  |
|  |  | Group 4 | 1 (Ref) | 1 (Ref) |  | 1 (Ref) | 1 (Ref) |  | 1 (Ref) | 1 (Ref) |
|  |  | Group 3 | 1.06 (0.98-1.15) | 1.10 (0.97-1.25) |  | 1.17 (0.81-1.68) | 1.22 (0.98-1.52) |  | 1.13 (0.83-1.55) | 0.77 (0.57-1.03) |
|  |  | Group 2 | 1.25 (1.14-1.37) | 1.17 (1.00-1.37) |  | 1.28 (0.83-1.96) | 1.23 (0.95-1.59) |  | 0.98 (0.68-1.42) | 1.03 (0.75-1.40) |
|  |  | Group 1 | 1.52 (1.37-1.68) | 1.58 (1.33-1.88) |  | 1.58 (0.98-2.52) | 1.27 (0.95-1.68) |  | 1.28 (0.88-1.86) | 0.98 (0.70-1.37) |
|  |  | Continuous (per SD) | 1.19 (1.14-1.23) | 1.18 (1.11-1.25) |  | 1.16 (0.97-1.38) | 1.09 (0.98-1.21) |  | 1.08 (0.95-1.24) | 1.02 (0.90-1.15) |
|  | Absolute HGS |  |  |  |  |  |  |  |  |  |
|  |  | Group 4 | 1 (Ref) | 1 (Ref) |  | 1 (Ref) | 1 (Ref) |  | 1 (Ref) | 1 (Ref) |
|  |  | Group 3 | 1.04 (0.96-1.13) | 1.08 (0.95-1.22) |  | 1.04 (0.72-1.50) | 1.11 (0.89-1.39) |  | 0.86 (0.63-1.17) | 0.80 (0.60-1.07) |
|  |  | Group 2 | 1.16 (1.05-1.28) | 1.07 (0.89-1.29) |  | 1.17 (0.73-1.90) | 1.14 (0.86-1.51) |  | 0.77 (0.53-1.11) | 0.92 (0.68-1.26) |
|  |  | Group 1 | 1.35 (1.21-1.50) | 1.27 (1.05-1.54) |  | 1.35 (0.82-2.22) | 1.09 (0.79-1.50) |  | 0.89 (0.62-1.29) | 0.84 (0.59-1.18) |
|  |  | Continuous (per SD) | 1.13 (1.08-1.17) | 1.11 (1.04-1.19) |  | 1.21 (1.01-1.45) | 1.05 (0.93-1.20) |  | 0.97 (0.85-1.11) | 0.97 (0.86-1.09) |

**Note:** Group 1= the first quartile (lowest); Group 2=the second quartile; Group 3= the third quartile; Group 4= reference=the fourth quartile; Dominant HGS: maximum HGS of dominant hand; Absolute HGS: the sum of the maximum HGS of both hands; Relative HGS: absolute HGS divided by BMI.

**Supplementary Table 4** Associations between baseline HGS and future stroke and heart disease using competing risk model

|  |  | **SHARE** | |  | **HRS** | |  | **CHARLS** | |
| --- | --- | --- | --- | --- | --- | --- | --- | --- | --- |
|  |  | HR (95% CI) | *P* |  | HR (95% CI) | *P* |  | HR (95% CI) | *P* |
| **Stroke** |  |  |  |  |  |  |  |  |  |
| Dominant HGS |  |  |  |  |  |  |  |  |  |
| Group 4 |  | Ref | - |  | Ref | - |  | Ref | - |
| Group 3 |  | 1.27 (1.14-1.41) | <0.001 |  | 1.23 (0.92-1.66) | 0.1648 |  | 0.90 (0.58-1.41) | 0.6418 |
| Group 2 |  | 1.31 (1.17-1.46) | <0.001 |  | 1.67 (1.26-2.22) | 0.0004 |  | 1.12 (0.73-1.72) | 0.6126 |
| Group 1 |  | 1.34 (1.19-1.50) | <0.001 |  | 1.58 (1.18-2.12) | 0.0025 |  | 2.10 (1.39-3.17) | 0.0005 |
| Continuous (per SD) |  | 1.11 (1.05-1.17) | <0.001 |  | 1.25 (1.09-1.44) | 0.0020 |  | 1.38 (1.16-1.63) | 0.0002 |
| Relative HGS |  |  |  |  |  |  |  |  |  |
| Group 4 |  | Ref | - |  | Ref | - |  | Ref | - |
| Group 3 |  | 1.19 (1.06-1.334) | 0.0028 |  | 1.34 (0.99-1.80) | 0.0586 |  | 1.24 (0.77-2.01) | 0.3768 |
| Group 2 |  | 1.37 (1.22-1.53) | <0.001 |  | 1.51 (1.13-2.02) | 0.0051 |  | 1.87 (1.21-2.90) | 0.0053 |
| Group 1 |  | 1.46 (1.30-1.64) | <0.001 |  | 1.61 (1.19-2.18) | 0.0021 |  | 2.05 (1.32-3.18) | 0.0013 |
| Continuous (per SD) |  | 1.18 (1.13-1.25) | <0.001 |  | 1.20 (1.05-1.38) | 0.0074 |  | 1.40 (1.17-1.67) | 0.0002 |
| Absolute HGS |  |  |  |  |  |  |  |  |  |
| Group 4 |  | Ref | - |  | Ref | - |  | Ref | - |
| Group 3 |  | 1.29 (1.11-1.51) | 0.0012 |  | 1.35 (0.99-1.83) | 0.0559 |  | 1.02 (0.66-1.58) | 0.9361 |
| Group 2 |  | 1.36 (1.18-1.56) | <0.001 |  | 1.60 (1.19-2.16) | 0.0019 |  | 1.16 (0.76-1.77) | 0.4955 |
| Group 1 |  | 1.55 (1.33-1.80) | <0.001 |  | 1.69 (1.24-2.29) | 0.0009 |  | 1.78 (1.17-2.70) | 0.0073 |
| Continuous (per SD) |  | 1.14 (1.08-1.20) | <0.001 |  | 1.24 (1.08-1.43) | 0.0025 |  | 1.32 (1.10-1.59) | 0.0033 |
| **Heart disease** |  |  |  |  |  |  |  |  |  |
| Dominant HGS |  |  |  |  |  |  |  |  |  |
| Group 4 |  | Ref | - |  | Ref | - |  | Ref | - |
| Group 3 |  | 1.09 (1.02-1.17) | 0.0139 |  | 1.33 (1.10-1.60) | 0.0030 |  | 0.87 (0.72-1.05) | 0.1539 |
| Group 2 |  | 1.11 (1.04-1.19) | 0.0025 |  | 1.17 (0.97-1.41) | 0.1029 |  | 0.89 (0.73-1.09) | 0.2656 |
| Group 1 |  | 1.12 (1.04-1.29) | 0.0028 |  | 1.22 (1.00-1.51) | 0.0563 |  | 0.90 (0.73-1.12) | 0.3364 |
| Continuous (per SD) |  | 1.02 (0.98-1.06) | 0.2956 |  | 1.09 (0.98-1.21) | 0.0995 |  | 0.98 (0.89-1.08) | 0.6931 |
| Relative HGS |  |  |  |  |  |  |  |  |  |
| Group 4 |  | Ref | - |  | Ref | - |  | Ref | - |
| Group 3 |  | 1.15 (1.07-1.24) | 0.0002 |  | 0.99 (0.81-1.19) | 0.8815 |  | 0.96 (0.78-1.18) | 0.6853 |
| Group 2 |  | 1.28 (1.19-1.38) | <0.0001 |  | 1.10 (0.91-1.33) | 0.3106 |  | 1.13 (0.93-1.38) | 0.2122 |
| Group 1 |  | 1.31 (1.22-1.42) | <0.0001 |  | 1.20 (0.99-1.46) | 0.0608 |  | 1.12 (0.91-1.38) | 0.2879 |
| Continuous (per SD) |  | 1.11 (1.08-1.15) | <0.0001 |  | 1.10 (1.00-1.21) | 0.0553 |  | 1.04 (0.95-1.14) | 0.4215 |
| Absolute HGS |  |  |  |  |  |  |  |  |  |
| Group 4 |  | Ref | - |  | Ref | - |  | Ref | - |
| Group 3 |  | 1.21 (1.10-1.33) | <0.001 |  | 1.19 (0.98-1.43) | 0.0743 |  | 0.89 (0.73-1.08) | 0.2387 |
| Group 2 |  | 1.21 (1.10-1.33) | <0.001 |  | 1.09 (0.90-1.32) | 0.3797 |  | 0.96 (0.79-1.16) | 0.6655 |
| Group 1 |  | 1.26 (1.14-1.39) | <0.001 |  | 1.16 (0.95-1.43) | 0.1506 |  | 0.88 (0.71-1.10) | 0.2582 |
| Continuous (per SD) |  | 1.02 (0.98-1.06) | 0.2661 |  | 1.06 (0.96-1.18) | 0.2531 |  | 0.97 (0.88-1.06) | 0.4520 |

**Note:** Group 1= the first quartile (lowest); Group 2=the second quartile; Group 3= the third quartile; Group 4= reference=the fourth quartile; Dominant HGS: maximum HGS of dominant hand; Absolute HGS: the sum of the maximum HGS of both hands; Relative HGS: absolute HGS divided by BMI.
